# From red to white urine: a patient's nightmare with a rather benign outcome

**DOI:** 10.1186/1471-2369-13-7

**Published:** 2012-02-01

**Authors:** Benjamin Knier, Beatrix Büschges-Seraphin, Karl F Hilgers, Kerstin U Amann, Michael Uder, Kai-Uwe Eckardt, Johannes Jacobi

**Affiliations:** 1 Department of Nephrology & Hypertension, University of Erlangen-Nuremberg, Ulmenweg 18, 91054 Erlangen, Germany; 2 KfH Dialysezentrum Fürth, Jakob-Henle-Haus, Robert-Koch str. 41, 90766 Fürth, Germany; 3 Department of Pathology, University of Erlangen-Nuremberg, Krankenhausstr. 12, 91054 Erlangen, Germany; 4 Department of Radiology, University of Erlangen-Nuremberg, Maximiliansplatz 1, 91054 Erlangen, Germany

**Keywords:** chyluria, thin basement membrane nephropathy

## Abstract

**Background:**

Chyluria is a medical condition with presence of chyle in the urine. The disease is most prevalent in endemic regions of Africa and the Indian subcontinent where it is mostly caused by parasitic infections, particularly lymphatic filariasis due to wucheria bancrofti. Non-parasitic chyluria, however, is a very rare finding.

**Case Presentation:**

We report the case of a 48 year old woman who developed a lymphorenal fistula with chyluria following ureterrenoscopy with biopsies taken for urological work-up of persistent macrohematuria. Renal biopsy confirmed the diagnosis of benign familial hematuria due to thin basement nephropathy, a condition frequently associated with episodes of macrohematuria.

**Conclusions:**

This case highlights a rare case of non-parasitic chyluria as a complication of urological work-up for macrohematuria of benign nature.

## Background

Chyluria is defined as the presence of lymphatic fluid within the urinary tract [1]. Patients suffering from chyluria typically present with milky and turbid urine, in particular after fatty meals. Urinalysis shows proteinuria which can exceed into nephrotic range while renal function is usually unaltered.

Most cases of chyluria are endemic and due to parasitic infections. The highest prevalence is found in tropical countries where up to 10% of patients with filariasis may experience chyluria [2]. In contrast, non-parasitic chyluria is a very rare condition most frequently seen in patients with malignancies, following surgical interventions or abdominal trauma or due to congenital malformations of the lymphatic system. The differential diagnosis of milky urine includes pyuria, lipiduria and phosphaturia.

Treatment of parasitic chyluria consists of potent microfilaricidal agents such as diethylcarbamazine [3]. The largely benign nature and spontaneous remission rates of up to 50% under diet restriction (low fat diet with medium-chain triglyceride supplementation) in non-parasitic chyluria favour a conservative management. Persistent chyluria, however, can lead to malnutrition due to loss of proteins and lipids [4]. Under such circumstances instillation of sclerosing agents such as silver nitrate solution may induce closure of lymphorenal fistulas with success rates as high as 80% [5]. However, severe complications such as necrotizing ureteritis and renal failure have been reported with this procedure [6,7]. Surgical options include retroperitoneoscopic or surgical renal pedicle lymphatic disconnection [8].

### Case Presentation

A 48-year-old woman was referred to our hospital due to turbid urine, preceded by a three-month lasting episode of macrohematuria.

The patient had suffered from painless macrohematuria in the period from June to September 2010. Onset of hematuria was spontaneous; the first bloody urine was recognized after physical exercise following a hiking tour in the mountains. Treatment with several antibiotics by her local physician did not improve her condition. In July 2010 she underwent urological examination including i.v. urography, cystoscopy and retrograde pyelogram. Computed tomography ruled out a malignant process or signs indicative for pyelonephritis or nephrolithiasis. Gynecological examination was normal. Since macrohematuria persisted, urological work-up was intensified. Bilateral ureterorenoscopy with biopsies taken from a suspicious mucosal lesion within the right lower renal calyx was performed. Histological examination of the specimen showed non-characteristic reactive changes. Invasive angiography of both kidneys excluded an arteriovenous fistula, aneurysm, angioma or vascular malformation.

In August 2010 she was referred to a nephrologist for further evaluation of macrohematuria. Microscopic urinalysis revealed significant hematuria with eumorphic red blood cells without evidence of akanthocytes or red cell casts. Urine sampling revealed nephrotic range proteinuria (14 g/day), urinary protein electrophoresis showed unselective glomerular proteinuria. Notably, the patient had no edema and was only mildly hypoproteinemic (serum albumine 34 g/l). By the end of September 2010, macrohematuria disappeared spontaneously. From this time on, the urine was milky and turbid, only the first morning urine was of normal appearance.

The patient's previous history yielded microhematuria during two pregnancies. During her first pregnancy she had mild arterial hypertension which resolved after delivery. At 28 years of age she underwent surgical treatment for recurrent sinusitis maxillaris. Otherwise, medical history was unremarkable and the patient was on no medication. She had not been outside Europe during the past years.

Vital signs upon presentation were as follows: blood pressure 140/90 mmHg, heart rate 72 beats/minute, respiratory rate 15/minute, temperature 36.5°Celsius. Physical examination was unremarkable; no edema or skin abnormalities were present. Initial laboratory testing was remarkable for hypoproteinemia (total protein 57.9 g/l), hypoalbuminemia (albumin 34.5 g/l) and mild hypercholesterolemia (total cholesterol 242 mg/dl). Whole blood count, serum creatinine (0.71 m/dl) and serum urea (25 mg/d) were normal.

**Figure 1:**
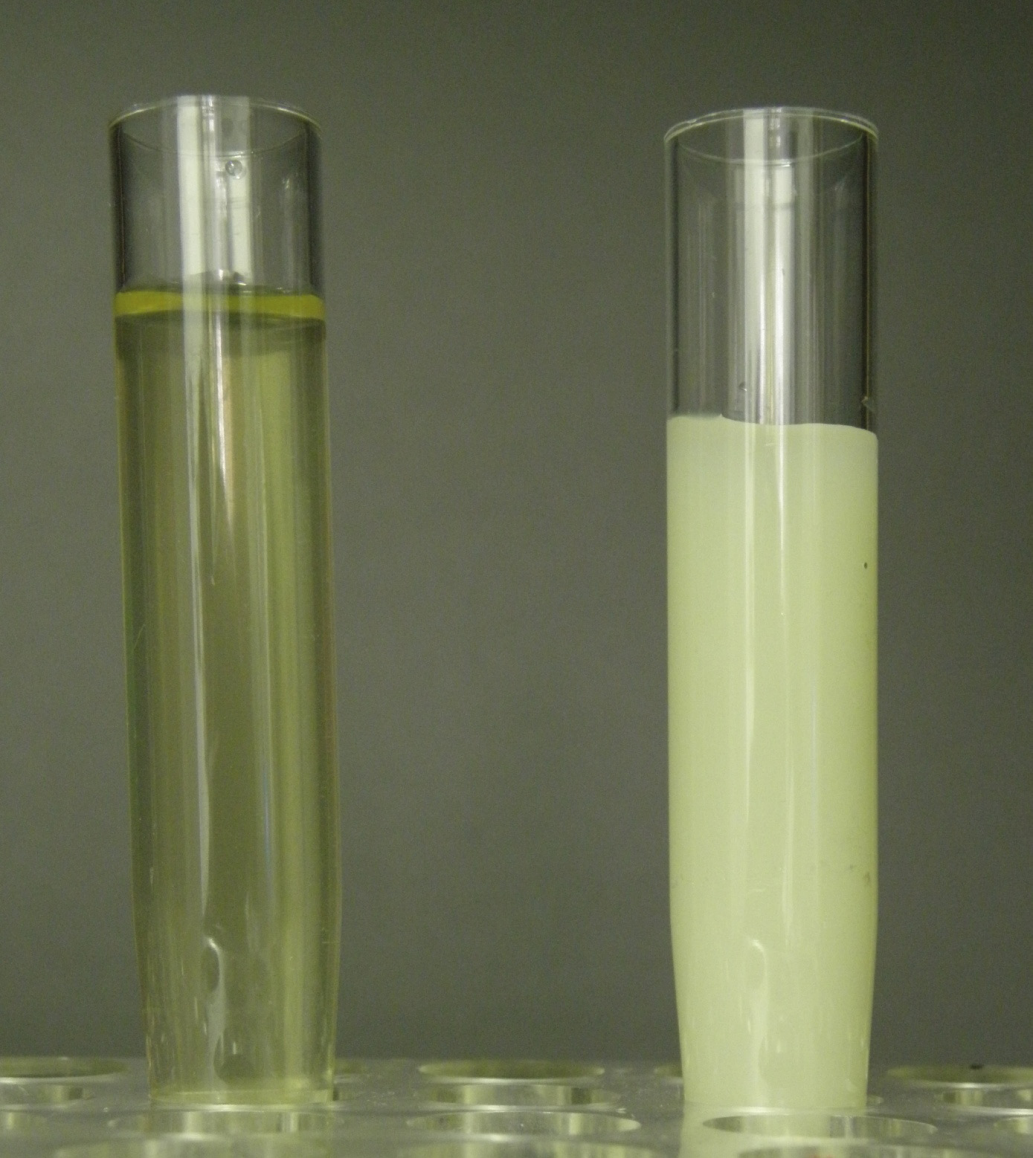
**Macroscopic appearance of an over-night fasting urine sample (left); postprandial urine sample 2 hours after breakfast (right)**.

The first morning urine was of normal appearance (Figure 1, left) and revealed normal ranges for urinary triglycerides (< 5 mg/dl) and albuminuria (24 mg/g creatinine). A postprandial urine sample taken two hours after breakfast was milky and turbid (Figure 1, right) and revealed increased urinary triglycerides (268 mg/dl) and marked albuminuria (3872 mg/g creatinine). Based on these findings the clinical diagnosis of chyluria was made.

**Figure 2:**
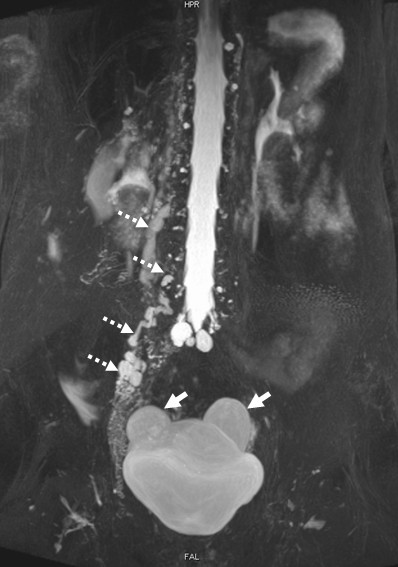
**Magnetic resonance imaging (T2 weighted HASTE sequence) showing bilateral ovary cysts (solid arrows) and a right-sided paravertebral lymphmalformation (dashed arrows)**.

Magnetic resonance imaging revealed bilateral ovary cysts and a right-sided lymphmalformation (Figure 2). The exact location of the lymphorenal/lymphoureteral fistula could not be determined. Since there was no immediate need for interventional therapy no further diagnostic work-up was performed and the patient was discharged and advised to maintain a low fat diet.

**Figure 3:**
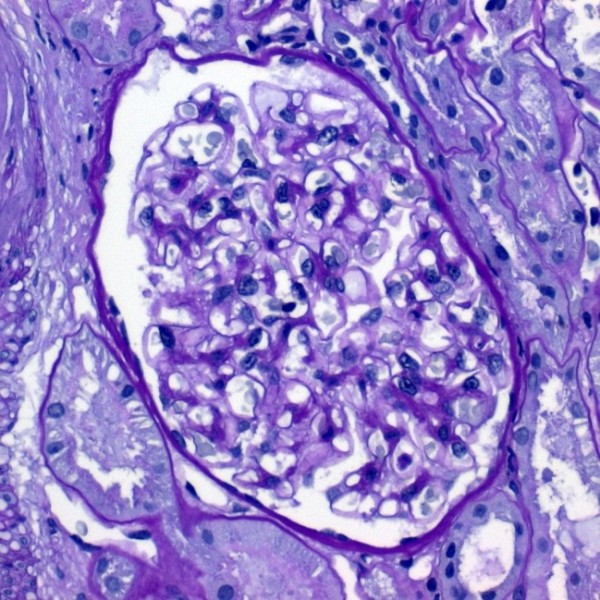
**Light microscopy (PAS stain) of renal biopsy showing mild mesangial matrix expansion**. Magnification × 40.

**Figure 4:**
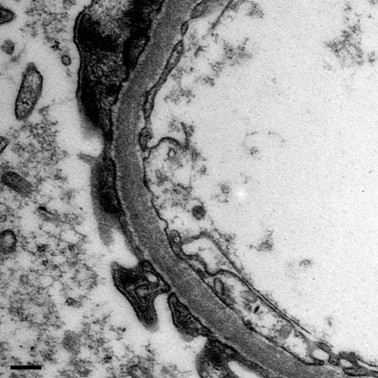
**Electron microscopy with marked thinning of the glomerular basement membrane**. Magnification: × 27800. Scale bar: 200 nm.

Prior to discharge a renal biopsy was performed to further clarify the patient's episodic macrohematuria. Light microscopy showed mild mesangial matrix expansion and mild arterio-arteriolosclerosis (Figure 3). Direct immunofluorescent staining was negative for immunoglobulines or complement ruling out any immune complex glomerulonephritis. Electron microscopy showed thin glomerular basement membranes (152-203 nm; Figure 4). Histological findings were compatible with the diagnosis of thin basement membrane nephropathy.

## Conclusions

In the current case report we describe a 48-year-old woman presenting with episodes of macrohematuria followed by chyluria. After profound urologic evaluation, the diagnoses of thin basement membrane nephropathy and chyluria were made. Thin basement membrane nephropathy, also known as benign familial hematuria, is a common cause of episodes of macrohematuria although most patients present with persistent or intermittent asymptomatic microscopic hematuria.

The disease which affects at least 1% of the general population belongs to the group of type IV collagen nephropathies [9]. Approximately 40% of patients display mutations with the COL4A3 or COL4A4 genes [9]. The disorder is non-progressive and despite hematuria patients do not usually present with proteinuria. Single episodes of macrohematuria are seen in 5-22% of all patients and are often triggered by physical exercise and/or hypertension as in our case [9].

In the present case the onset of turbid and milky urine, identified as chyluria, was first noticed after cessation of the episode of macrohematuria. Since radiographic findings suggested a right-sided, benign congenital lymphmalformation it seems likely that aberrant lymphatic vessels led to a lymphorenal/lymphoureteral fistula following ureterrenoscopy with biopsy taking.

Since the patient denied further evaluation to identify the exact location of the fistula such as lymphangiography or cystoscopy/ureterrenoscopy after a fatty meal, we informed the patient of the benign nature of chyluria with a good likelihood of a spontaneous remission under a low fat diet.

Unfortunately, until now asymptomatic chyluria persists, but the patient is feeling well. Since persistent chyluria can cause malnutrition due to excessive proteinuria the patient is seen on a regular basis in our outpatient clinic. At present proteinuria is in the range of 2.5 g/l (spot urine at outpatient clinic ~2 h after breakfast).

In summary, we present a rare case of chyluria following an episode of macrohematuria with extensive invasive urologic diagnostic work-up. This case highlights the importance of a cautious evaluation of patients presenting with macrohematuria. An early interdisciplinary approach involving both urologists as well as nephrologists may be useful to avoid unnecessary diagnostic procedures and subsequent complications.

## Consent

Written informed consent was obtained from the patient for publication of this case.

## Competing interests

The authors declare that they have no competing interests.

## Authors' contributions

BK, BBS, JJ, KUE and KFH were the treating physicians of the patient reported. KA performed the evaluation of the renal biopsy. MU performed the radiographic studies. The manuscript was prepared by BK and JJ, all authors participated in the discussion of the manuscript and approved the final version.

## Pre-publication history

The pre-publication history for this paper can be accessed here:

http://www.biomedcentral.com/1471-2369/13/7/prepub
